# Burden and trends of infectious disease mortality attributed to air pollution, unsafe water, sanitation, and hygiene, and non-optimal temperature globally and in different socio-demographic index regions

**DOI:** 10.1186/s41256-024-00366-x

**Published:** 2024-06-28

**Authors:** Qiao Liu, Jie Deng, Wenxin Yan, Chenyuan Qin, Min Du, Yaping Wang, Shimo Zhang, Min Liu, Jue Liu

**Affiliations:** 1 Department of Epidemiology and Biostatistics, School of Public Health, Peking University, No. 38, Xueyuan Road, Haidian District, Beijing, 100191 China; 2 Key Laboratory of Epidemiology of Major Diseases, Ministry of Education, Peking University, Haidian District, Beijing, China; 3 Institute for Global Health and Development, Peking University, Haidian District, Beijing, China; 4 Global Center for Infectious Disease and Policy Research & Global Health and Infectious Diseases Group, Peking University, Beijing, China; 5 Institute of Environmental Medicine, Peking University, Beijing, China

## Abstract

**Background:**

Environmental factors greatly impact infectious disease-related mortality, yet there's a lack of comprehensive global studies on the contemporary burden and trends. This study aims to evaluate the global burden and trends of infectious disease mortality caused by air pollution, unsafe water, poor sanitation, and non-optimal temperature across Socio-Demographic Index (SDI) regions from 1990 to 2019.

**Methods:**

This observational study utilized data from the Global Burden of Diseases Study to examine mortality rates from infectious diseases attributed to environmental risk factors between 1990 and 2019, including air pollution, unsafe water, sanitation, handwashing facilities (UWSH), and non-optimal temperatures. Age-standardized mortality rates (ASMRs) and estimated annual percentage change (EAPC) were utilized to present infectious disease mortality, and its trajectory influenced by environmental risk factors over the years. Nonlinear regression was conducted to explore the association between the SDI and ASMRs across regions from 1990 to 2019.

**Results:**

In 2019, global infectious disease deaths linked to air pollution, UWSH, and non-optimal temperature reached a startling 2,556,992. Disease mortality varied widely across SDI regions, with the highest number of deaths due to air pollution and UWSH in Low SDI regions, and deaths from non-optimal temperature primarily in High SDI regions. Age disparities emerged, with children under five and the elderly most affected. However, an increasing mortality trend was observed among seniors (65–69, 75–79, and over 80) in High SDI regions due to enteric infections linked to UWSH. Globally, a consistent decrease in ASMR was seen from 1990 to 2019 for all diseases connected to these factors, except for respiratory infections linked to non-optimal temperature.

**Conclusions:**

Our study underscores the significant impact of air pollution, UWSH, and non-optimal temperatures on global infectious disease mortality, particularly among vulnerable groups such as children and the elderly. It's important to tackle these challenges with targeted interventions aiming to enhance environmental quality, improve water and sanitation systems, and control extreme temperatures. In addition, international cooperation is essential for bridging regional disparities and driving global public health initiatives forward, thereby helping achieve Sustainable Development Goals more effectively.

## Introduction

Human well-being is significantly impacted by various environmental factors. According to the World Health Organization (WHO), in 2016, 13.7 million deaths, constituting 24% of global mortality, were attributed to modifiable environmental risks, underscoring that nearly one in four global deaths was linked to environmental conditions [1]. Specifically, air pollution, a contributor to both communicable and noncommunicable diseases, was responsible for approximately one in eight deaths [2]. Furthermore, cholera, primarily transmitted through faecally contaminated water or food, has affected 47 countries, with an annual report of about 2.9 million cases [2]. Additionally, inadequate water, sanitation, and hygiene lead to 829 thousand preventable deaths from diarrheal diseases each year, including 297 thousand deaths of children aged 5 and under [2]. It was also estimated that 1.69 million deaths were attributable to non-optimal temperature globally in 2019 [3]. These compelling statistics underscore the imperative to recognize and address the pervasive impact of environmental factors on health. The emphasis on the profound influence of environmental factors on global mortality rates necessitates increased awareness and concerted efforts to foster healthier living environments worldwide.

The impact of environmental factors on health is prominently demonstrated in the domain of infectious diseases. Environmental conditions play crucial roles in disease transmission and prevalence, shaping patterns that significantly contribute to global morbidity and mortality. Recent reports have highlighted the close association between emerging infectious diseases and environmental factors, particularly the rise in diseases originating from wildlife [4]. Previous studies have validated a correlation between common air pollutants and death from lower respiratory infections, revealing that nitrogen dioxide (per 10 μg/m^3^) and black carbon (per 0.5 10^−5^ m^−1^) are linked to a 10% to 12% increase in combined mortality from pneumonia and influenza [5]. In contrast to air pollution, which primarily leads to noncommunicable disease-related deaths, illnesses resulting from unsafe drinking water predominantly manifest as infectious diseases, including diarrhea and parasitic infections. Prior research has demonstrated that interventions to improve drinking water, sanitation, and hygiene can effectively reduce the incidence of childhood diarrhea in low- and middle-income countries [6–9]. However, despite these findings, as of 2020, 2.0 billion people lacked access to safely managed drinking water services, 3.6 billion lacked access to safely managed sanitation services, and 2.3 billion lacked access to handwashing facilities with soap and water at home [10]. Moreover, climate and weather significantly influence the duration, timing, and intensity of disease outbreaks, reshaping the global landscape of infectious diseases [11]. For example, a study in China has revealed that each 5 °C increase in average temperature above 10 °C was associated with a 22% (95% CI, 17% to 28%) increase in malaria cases [12]. Furthermore, the potential expansion of pathogen or vector ranges due to climate change, coupled with increased global connectivity, can potentially facilitate a faster dissemination of pathogens into new areas [13].

Considering the paramount significance of environmental factors and their profound influence on global welfare, they emerge as vital touchstones within the framework set by the Sustainable Development Goals (SDGs). Notably, they feature prominently in Goal 3 – “Ensure healthy lives and promote well-being for all at all ages”, Goal 6 – “Ensure availability and sustainable management of water and sanitation for all”, and Goal 13 – “Take urgent action to combat climate change and its impacts” [14]. In this context, the intersection of environmental factors and infectious diseases becomes a critical focus of inquiry. However, a noticeable dearth exists in global-scale studies examining the contemporary burden and trends of infectious diseases attributed to environmental factors, representing a significant gap hindering the realization of SDGs.

This study, acknowledging the existing research void, aims to shed light on the global burden and trends in infectious disease mortality linked to certain environmental factors, focusing on different Socio-Demographic Index (SDI) regions from 1990 to 2019. Through a meticulous exploration of this inquiry, our findings have the potential to offer valuable insights into the evolving landscape of infectious diseases influenced by certain environmental factors and the regional variations therein. This contribution seeks to enhance our nuanced understanding of the intricate interplay between global health and environmental conditions, ultimately contributing to the fulfillment of SDGs.

## Methods

### Study design

This was an observational study using data obtained from the 2019 Global Burden of Disease study (GBD 2019) result tools. The GBD results, a comprehensive regional and global research program encompassing hundreds of diseases, injuries, and risk factors, allows researchers access to a wealth of global health data [15]. This observational study capitalized on this extensive dataset to analyze and draw conclusions about particular health concerns.

### Variables

The number of deaths with its 95% uncertainty interval (UI) per year, and their mortality rates with 95% UIs of infectious diseases attributed to environmental risk factors were extracted. Infectious diseases included enteric infections (diarrheal diseases, specifically referring to infectious diarrheal diseases), respiratory infections (lower respiratory infections, upper respiratory infections, and otitis media), and other infectious diseases (encephalitis and meningitis). Environmental risk factors in this study included air pollution (ambient particulate matter pollution and household air pollution from solid fuels), non-optimal temperature (high temperature and low temperature), and unsafe water, sanitation, and handwashing (UWSH) (unsafe water source, unsafe sanitation, and no access to handwashing facility). The Socio-demographic Index (SDI) of 21 GBD regions from 1990 to 2019 was also extracted from the GBD 2019 result tools. The SDI is a composite indicator of development status strongly correlated with health outcomes. It is the geometric mean of 0 to 1 indices of total fertility rate under the age of 25, mean education for those ages 15 and older, and lag distributed income per capita. As a composite, a country with an SDI of 0 would have a theoretical minimum level of development relevant to health, while a country with an SDI of 1 would have a theoretical maximum level. Low SDI was between 0 and 0.455, Low-middle SDI was between 0.455 and 0.608, Middle SDI was between 0.608 and 0.690, High-middle SDI was between 0.690 and 0.805, and High SDI was between 0.805 and 1 [16].

### Data collection and processing

Data was gathered from the GBD 2019 result tools, established by the GBD group [15]. The general methodological approaches to estimate the mortality were described elsewhere [17]. Briefly, all available data on causes of death and exposure of risk factors were standardized and pooled into a single database used to generate cause-specific estimates by age, sex, year, and geography; then multiple models, such as cause of death ensemble modelling, disease model-Bayesian meta-regression, comorbidity correction and so on were used to estimate comparable data of different diseases across the world [17]. Furthermore, GBD study used a model to link the death rates of infectious diseases to environmental risk factors. The GBD measured how people are exposed to these risks, how these exposures are related to health outcomes, and the percentage of deaths that would be prevented by reducing exposure to these risks. This approach, however, may vary, and captures uncertainty regarding the quality of data and the certitude of the used models [18].

We reported the death results of infectious diseases attributed to air pollution, UWSH, and non-optimal temperature, in five SDI regions (High, High-middle, Middle, Low-middle, and Low SDI regions) and the global total data from 1990 to 2019, and arranged incidence and death data into successive 5-year age intervals from < 5 years to 75–79 years, plus the 80 + years group.

### Statistical analysis

The absolute number of deaths represented the actual impact of infectious disease mortality attributed to environmental risk factors in each SDI region and at global level, and its relative change was defined as $\frac{{Number}_{2019}-{Number}_{1990}}{{Number}_{1990}}\times 100\%$, which showed the overall change between 1990 and 2019. Age-standardized mortality rate (ASMR), which were directly extracted from the GBD result tool, [15] were calculated by applying the age-specific rates to a GBD World Standard Population, and were used to compare populations with different age structures or for the same population over time in which the age profiles changed accordingly.

The Estimated Annual Percentage Change (EAPC) is a commonly used tool to quantified the rate trend over a specific interval [19, 20]. A regression line was fitted to the natural logarithm of the rates (*y* = *α* + *βx* + *ε*, where y = ln(rate) and x = calendar year). EAPC was calculated as $({e}^{\beta }-1)\times 100\%$, with 95% confidence intervals (CIs) obtained from the linear regression model. In this study, overall EAPC was calculated by the annual ASMR of each category of infectious diseases attributed to environmental risk factors in five SDI regions and at global level, and EAPC in different age groups was calculated by the age-specific mortality rate. The term “increase” was used to describe trends when the EAPC and its lower boundary of 95% CI were both > 0. In contrast, “decrease” was used when the EAPC and its upper boundary of 95% CI, were both < 0. Otherwise, the term “stable” was used.

The population attributable fraction (PAF), which represents the proportion of risk that would be reduced in a given year if the exposure to a risk factor in the past were reduced to an ideal exposure scenario [17]. In this study, we extracted mortality data for all causes of infectious diseases and calculated the PAF of certain risk factors for certain infectious diseases in each year by the formula: $\frac{death\;number\;attributed\;to\;certain\;risk\;factors}{death\;number\;by\;all\;causes}\times100\%$.

Finally, we conducted a non-linear regression (second order polynomial) to explore the association between SDI and ASMRs in 21 GBD regions throughout 1990 to 2019. A regression curve was fitted to the ASMR (*y* = *α* + *βx* + *γx*^*2*^, where y = the value of ASMRs and x = SDI).

All the statistical analyses were conducted using the R program (version 4.4.1).

## Results

### Burden and trends of infectious disease mortality attributed to air pollution, UWSH, and non-optimal temperature from 1990 to 2019

In 2019, global infectious disease deaths attributed to environmental risk factors reached a staggering 2,556,992, with contributions from air pollution, UWSH, and non-optimal temperature of 763,291, 1,656,887, and 245,814 deaths, respectively. The predominant cause of infectious disease deaths associated with air pollution was respiratory infections, contributing to 749,254 fatalities globally in 2019 (95% UI, 573,848 to 959,290). UWSH primarily resulted in deaths from enteric infections, while non-optimal temperature mainly caused respiratory infections, with 1,386,769 (95% UI, 978,063 to 2,009,500) and 245,814 (95% UI, 174,760 to 342,302) deaths, respectively, in 2019.

Examining different SDI regions in 2019, the highest number of infectious disease deaths attributed to air pollution and UWSH occurred in the Low SDI region, with 330,074 (95% UI, 250,942 to 423,208) respiratory infections attributed to air pollution, 6,546 (95% UI, 5,096 to 8,419) enteric infections attributed to air pollution, 2,529 (95% UI, 1,962 to 3,317) deaths from other infectious diseases attributed to air pollution, 640,329 (95% UI, 480,412 to 858,264) enteric infections attributed to UWSH, and 130,090 (95% UI, 58,429 to 200,590) respiratory infections attributed to UWSH. However, in the case of respiratory infections attributed to non-optimal temperatures, the highest death toll was observed in High SDI regions, with 69,216 (95% UI, 51,479 to 88,585) deaths in 2019 (Table 1).

**Table 1:** Burden and trends between 1990 and 2019 of infectious disease mortality attributed to air pollution, UWSH, and non-optimal temperature globally and in SDI regions

Characteristic	Death Number (95% UI)			Age-standardized Mortality Rate (per 100,000 population) (95% UI)		
	1990	2019	change (%)	1990	2019	EAPC (%)
***Air pollution & Respiratory infections***						
Global	1,504,928 (1,169,301 to 1,912,263)	749,254 (573,848 to 959,290)	-50.21	27.71 (21.66 to 34.76)	10.38 (7.89 to 13.28)	-3.4 (-3.49 to -3.31)
High SDI	21,892 (11,087 to 36,723)	23,083 (13,238 to 37,196)	5.44	2.2 (1.12 to 3.66)	1.05 (0.61 to 1.67)	-2.85 (-3.06 to -2.64)
High-middle SDI	82,249 (57,766 to 109,211)	39,568 (25,990 to 56,923)	-51.89	8.63 (6.05 to 11.39)	2.25 (1.48 to 3.23)	-4.69 (-4.87 to -4.52)
Middle SDI	342,721 (254,948 to 437,989)	117,768 (81,193 to 160,046)	-65.64	24.19 (18.02 to 30.49)	6.09 (4.2 to 8.3)	-4.61 (-4.7 to -4.53)
Low-middle SDI	570,717 (450,729 to 723,680)	238,282 (177,572 to 303,310)	-58.25	50.43 (39.83 to 62.41)	18.09 (13.52 to 23.06)	-3.53 (-3.65 to -3.42)
Low SDI	486,622 (363,491 to 652,712)	330,074 (250,942 to 423,208)	-32.17	83.65 (64.7 to 107.32)	40.74 (31.72 to 50.7)	-2.55 (-2.68 to -2.43)
***Air pollution & Enteric infections***						
Global	48,498 (34,102 to 61,101)	10,386 (8296 to 13,009)	-78.58	0.74 (0.52 to 0.93)	0.16 (0.13 to 0.2)	-5.2 (-5.52 to -4.87)
High SDI	17 (9 to 31)	2 (2 to 3)	-86.49	0 (0 to 0.01)	0 (0 to 0)	-5.29 (-6.04 to -4.52)
High-middle SDI	641 (472 to 828)	62 (43 to 85)	-90.38	0.06 (0.05 to 0.08)	0.01 (0.01 to 0.01)	-6.86 (-7.09 to -6.64)
Middle SDI	6328 (4893 to 7611)	795 (577 to 1079)	-87.44	0.31 (0.24 to 0.37)	0.05 (0.03 to 0.06)	-6 (-6.32 to -5.67)
Low-middle SDI	22,783 (14,827 to 30,699)	2974 (2268 to 3870)	-86.95	1.27 (0.82 to 1.71)	0.18 (0.13 to 0.23)	-6.41 (-6.71 to -6.1)
Low SDI	18,704 (13,293 to 23,503)	6546 (5096 to 8419)	-65	1.67 (1.19 to 2.1)	0.36 (0.28 to 0.47)	-5.22 (-5.52 to -4.92)
***Air pollution & Other infectious diseases***						
Global	7863 (6753 to 9328)	3651 (2907 to 4686)	-53.57	0.12 (0.1 to 0.14)	0.06 (0.04 to 0.07)	-2.47 (-2.85 to -2.09)
High SDI	17 (13 to 20)	4 (3 to 4)	-78.39	0 (0 to 0)	0 (0 to 0)	-4.63 (-4.77 to -4.5)
High-middle SDI	222 (191 to 261)	32 (25 to 41)	-85.78	0.02 (0.02 to 0.03)	0 (0 to 0.01)	-5.55 (-5.86 to -5.24)
Middle SDI	1113 (922 to 1349)	264 (207 to 333)	-76.33	0.05 (0.04 to 0.07)	0.02 (0.01 to 0.02)	-4.08 (-4.45 to -3.7)
Low-middle SDI	2557 (2085 to 3113)	820 (634 to 1074)	-67.91	0.14 (0.12 to 0.17)	0.05 (0.04 to 0.06)	-3.31 (-3.73 to -2.88)
Low SDI	3948 (3336 to 4848)	2529 (1962 to 3317)	-35.95	0.35 (0.3 to 0.43)	0.14 (0.11 to 0.18)	-2.97 (-3.25 to -2.68)
***UWSH & Enteric infections***						
Global	2,760,787 (2,122,261 to 3,452,311)	1,386,769 (978,063 to 2,009,500)	-49.77	55.49 (41.19 to 72.04)	18.94 (13.59 to 26.96)	-3.73 (-3.85 to -3.6)
High SDI	3647 (2407 to 5163)	3410 (2195 to 5090)	-6.49	0.5 (0.32 to 0.72)	0.16 (0.11 to 0.24)	-2.77 (-3.32 to -2.21)
High-middle SDI	61,698 (48,991 to 77,185)	19,403 (12,067 to 29,829)	-68.55	6.18 (4.89 to 7.73)	1.22 (0.79 to 1.77)	-5.66 (-5.8 to -5.52)
Middle SDI	450,105 (370,504 to 554,123)	160,231 (101,848 to 244,158)	-64.4	34.7 (26.16 to 47.08)	8.15 (5.2 to 12.26)	-4.84 (-4.95 to -4.74)
Low-middle SDI	1,268,310 (932,425 to 1,632,184)	562,788 (352,842 to 896,136)	-55.63	166.53 (111.04 to 230.39)	46.72 (28.61 to 75.65)	-4.36 (-4.45 to -4.26)
Low SDI	975,778 (721,568 to 1,229,660)	640,329 (480,412 to 858,264)	-34.38	235.48 (149.39 to 325.7)	88.32 (60.54 to 129.77)	-3.56 (-3.69 to -3.43)
***UWSH & Respiratory infections***						
Global	461,624 (199,438 to 718,070)	270,118 (119,526 to 421,044)	-41.49	8.44 (3.63 to 12.97)	3.74 (1.66 to 5.83)	-2.82 (-2.93 to -2.72)
High SDI	4299 (1695 to 7062)	5582 (2171 to 9393)	29.84	0.43 (0.17 to 0.71)	0.25 (0.1 to 0.42)	-2.16 (-2.28 to -2.04)
High-middle SDI	22,406 (9401 to 35,677)	10,035 (4099 to 16,465)	-55.21	2.33 (0.98 to 3.74)	0.58 (0.23 to 0.94)	-4.85 (-4.92 to -4.77)
Middle SDI	87,735 (37,339 to 138,487)	37,117 (15,810 to 59,550)	-57.69	6.3 (2.66 to 9.9)	1.92 (0.82 to 3.09)	-3.94 (-4.01 to -3.86)
Low-middle SDI	172,690 (73,823 to 266,384)	87,106 (38,882 to 136,039)	-49.56	15.41 (6.61 to 23.59)	6.57 (2.95 to 10.29)	-2.91 (-3 to -2.81)
Low SDI	174,253 (76,909 to 271,485)	130,090 (58,429 to 200,590)	-25.34	30.33 (13.64 to 46.02)	16.2 (7.32 to 24.93)	-2.26 (-2.35 to -2.16)
***Non-optimal temperature & Respiratory infections***						
Global	359,594 (257,631 to 608,180)	245,814 (174,760 to 342,302)	-31.64	7.67 (5.65 to 12.1)	3.41 (2.42 to 4.77)	-3.04 (-3.14 to -2.93)
High SDI	51,959 (38,855 to 67,443)	69,216 (51,479 to 88,585)	33.21	5.16 (3.86 to 6.7)	3.03 (2.28 to 3.85)	-2.34 (-2.53 to -2.15)
High-middle SDI	47,530 (34,783 to 62,225)	41,017 (28,315 to 55,519)	-13.7	5.1 (3.75 to 6.63)	2.27 (1.58 to 3.06)	-2.92 (-3.03 to -2.8)
Middle SDI	91,968 (69,510 to 133,785)	38,665 (25,473 to 57,769)	-57.96	6.35 (4.75 to 9.43)	2.01 (1.33 to 3)	-4.07 (-4.22 to -3.91)
Low-middle SDI	99,923 (64,883 to 200,881)	50,472 (31,163 to 76,884)	-49.49	8.71 (5.56 to 17.86)	3.85 (2.37 to 5.86)	-3.05 (-3.2 to -2.9)
Low SDI	68,121 (36,346 to 167,242)	46,374 (22,767 to 97,094)	-31.92	11.08 (6.01 to 27.74)	5.18 (2.67 to 10.29)	-2.81 (-2.94 to -2.69)

*SDI* Socio-Demographic Index, *EAPC* Estimated Annual Percentage Change, *UWSH* Unsafe water, sanitation, and handwashing

**Fig. 1:**
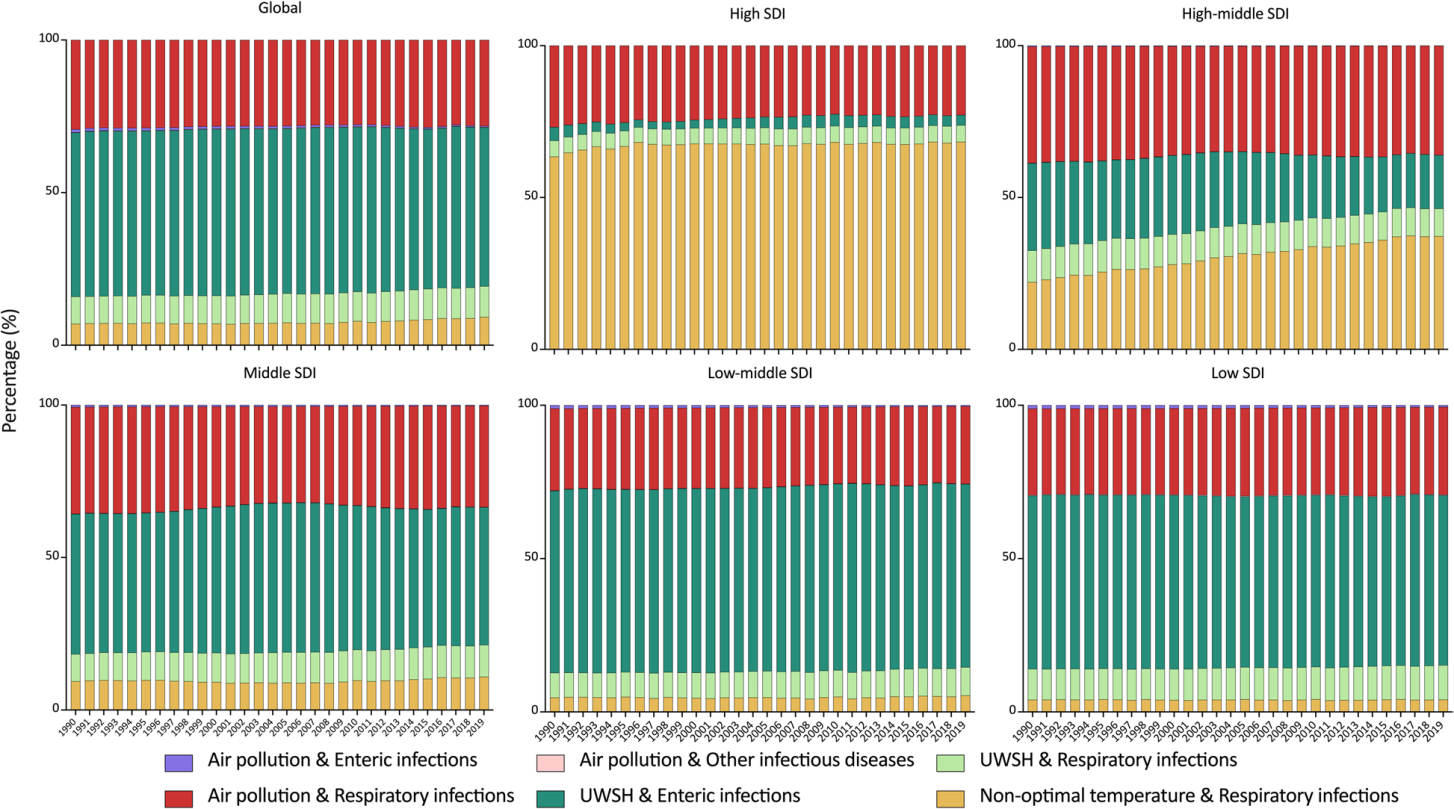
Proportions of deaths of infectious diseases attributed to air pollution, UWSH, and non-optimal temperature from 1990 to 2019, globally and by SDI regions. SDI, Socio-Demographic Index; UWSH, unsafe water, sanitation, and handwashing

On a global scale, enteric infections, attributed to UWSH, were responsible for the highest number of deaths from 1990 to 2019, ranging from 52.01% to 54.67%. This trend persisted in Middle, Low-middle, and Low SDI regions, where enteric infections attributed to UWSH constituted 44.88% to 49.03%, 59.35% to 61.57%, and 55.10% to 56.89% of all deaths, respectively, from 1990 to 2019. Conversely, in the High-middle SDI region, respiratory infections attributed to air pollution dominated deaths from 1990 to 2015 (34.65% to 38.30%). Subsequently, respiratory infections attributed to non-optimal temperature became the primary cause of deaths from 2016 to 2019, accounting for over 37%. Moreover, in the High SDI region, respiratory infections attributed to non-optimal temperature constituted more than 60% of deaths from 1990 to 2019 (Fig. 1).

Among the three categories of infectious diseases tied to air pollution, respiratory infections had the highest ASMR both in 1990 (27.71 per 100,000 population, 95% UI, 21.66 to 34.76) and 2019 (10.38 per 100,000 population, 95% UI, 7.89 to 13.28). UWSH and non-optimal temperature primarily resulted in enteric infections and respiratory infections, with ASMRs of 18.94 (95% UI, 13.59 to 26.96) and 3.41 (95% UI, 2.42 to 4.77) per 100,000 population in 2019 (Table 1).

All infectious diseases attributed to air pollution, UWSH, and non-optimal temperature exhibited declining trends in ASMR globally from 1990 to 2019, with the most rapid decrease observed in enteric infections attributed to air pollution (EAPC = -5.20%, 95% CI, -5.52% to -4.78%). This category decreased from 0.74 (95% CI, 0.52 to 0.93) per 100,000 population in 1990 to 0.16 (95% CI, 0.13 to 0.20) per 100,000 population in 2019. Following closely were enteric infections attributed to UWSH, exhibiting an average annual decrease of 3.73% (95% CI, 3.60% to 3.85%), declining from 8.44 (95% UI, 3.63 to 12.97) per 100,000 in 1990 to 3.74 (95% UI, 1.66 to 5.83) per 100,000 in 2019.

**Fig. 2:**
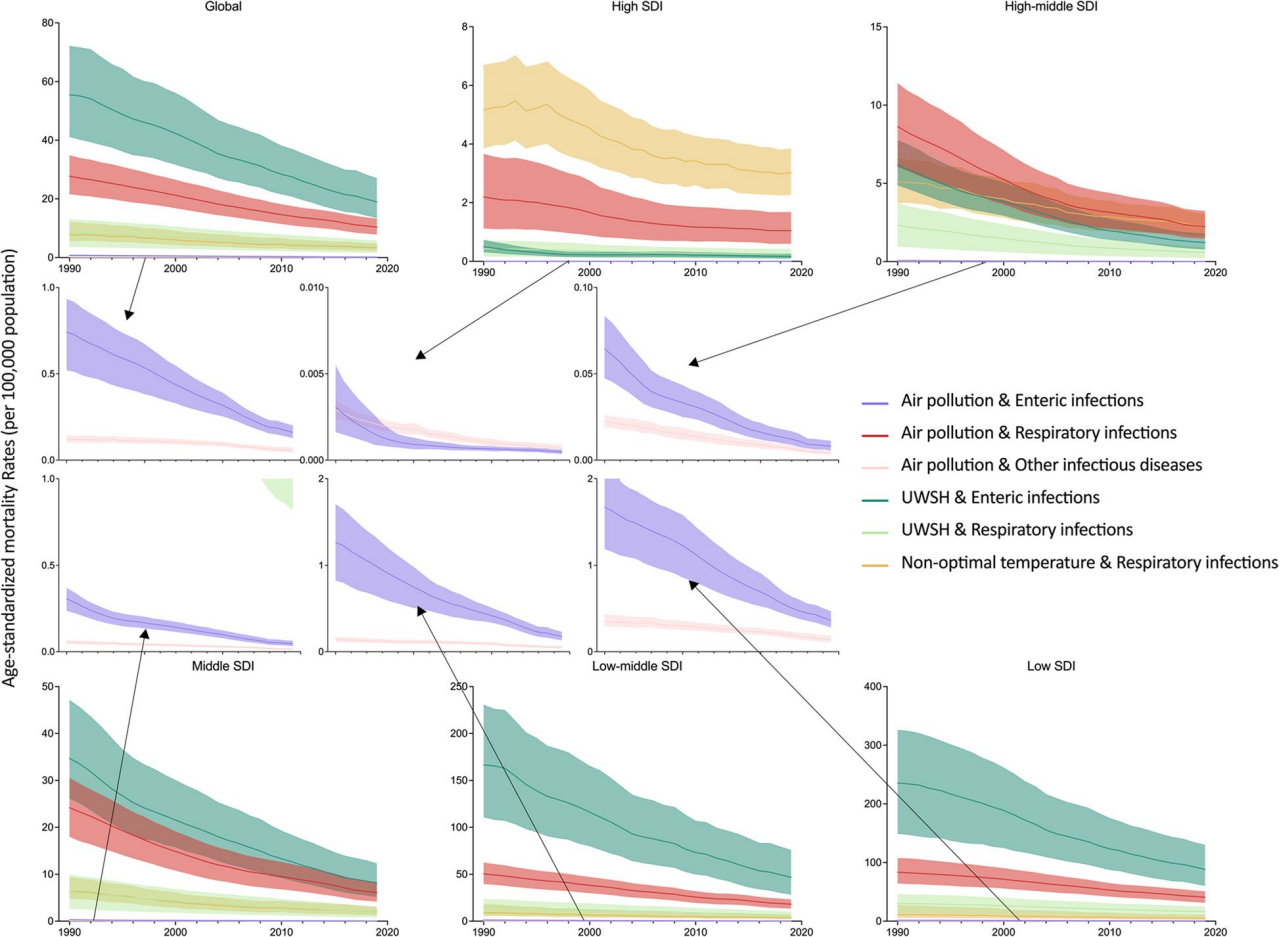
Trends of ASMRs of infectious diseases attributed to air pollution, UWSH, and non-optimal temperature from 1990 to 2019, globally and by SDI regions. ASMR, Age-standardized mortality rate; SDI, Socio-Demographic Index; UWSH, unsafe water, sanitation, and handwashing

Analyzing within different SDI regions, the highest ASMRs of infectious diseases associated with air pollution, UWSH, and non-optimal temperature were consistently observed in Low SDI regions, in both 1990 and 2019. Across all SDI regions, the ASMRs of infectious diseases attributed to air pollution, UWSH, and non-optimal temperature demonstrated declining patterns from 1990 to 2019 (all *p* < 0.05) (Fig. 2).

### Age disparities of infectious disease mortality attributed to air pollution, UWSH, and non-optimal temperature

**Fig. 3:**
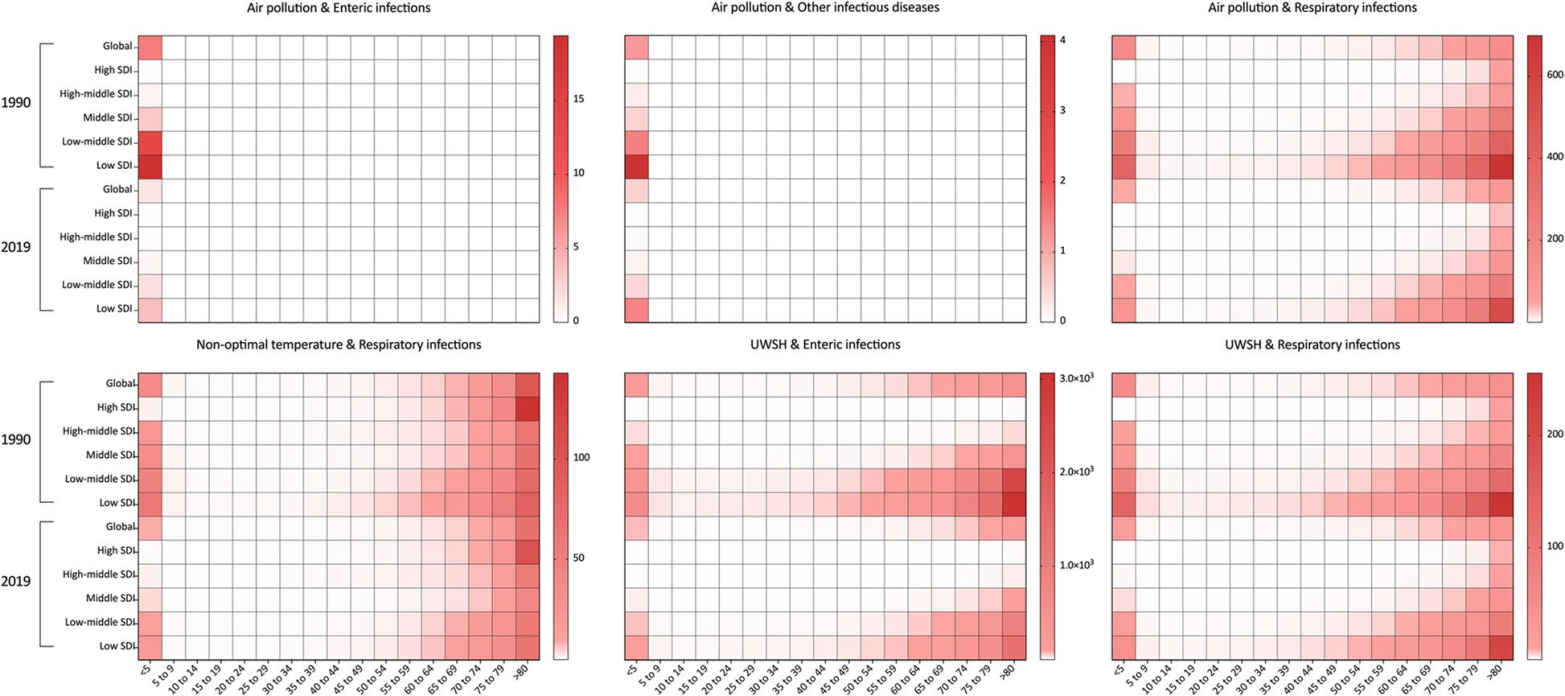
Mortality of infectious diseases attributed to air pollution, UWSH, and non-optimal temperature in different age groups, in 1990 and 2019, globally and by SDI regions. SDI, Socio-Demographic Index; UWSH, unsafe water, sanitation, and handwashing

Figure 3 illustrated the global mortality rates of infectious diseases that are attributable to air pollution, UWSH, and non-optimal temperature, in various SDI regions, and across different age groups for the years 1990 and 2019. Notably, air pollution was exclusively linked with deaths from enteric infections and other infectious diseases only in children under the age of 5, with this correlation not being observed in other age groups. The highest mortality rates were observed in the Low SDI region in 2019: 3.83 (95% UI, 2.98 to 4.93) per 100,000 for enteric infections and 1.48 (95% UI, 1.15 to 1.94) per 100,000 for other infectious diseases.

In cases of respiratory infections attributed to air pollution, non-optimal temperature, UWSH, and enteric infections due to UWSH, both children under five years old and the elderly were significantly impacted. In 2019, the global mortality rates for these conditions in children under 5 were 45.90 (95% UI, 33.50 to 60.41), 8.69 (95% UI, 4.90 to 16.17), 17.24 (95% UI, 7.78 to 27.39), and 71.02 (95% UI, 54.77 to 92.29) per 100,000 population, respectively. In individuals aged over 80, the rates were 101.96 (95% UI, 74.03 to 135.37), 66.46 (95% UI, 49.13 to 87.47), 33.08 (95% UI, 14.11 to 52.85), and 202.05 (95% UI, 116.59 to 330.79) per 100,000 population, respectively. Low SDI regions consistently reported the highest mortality rates across all age groups.

**Fig. 4:**
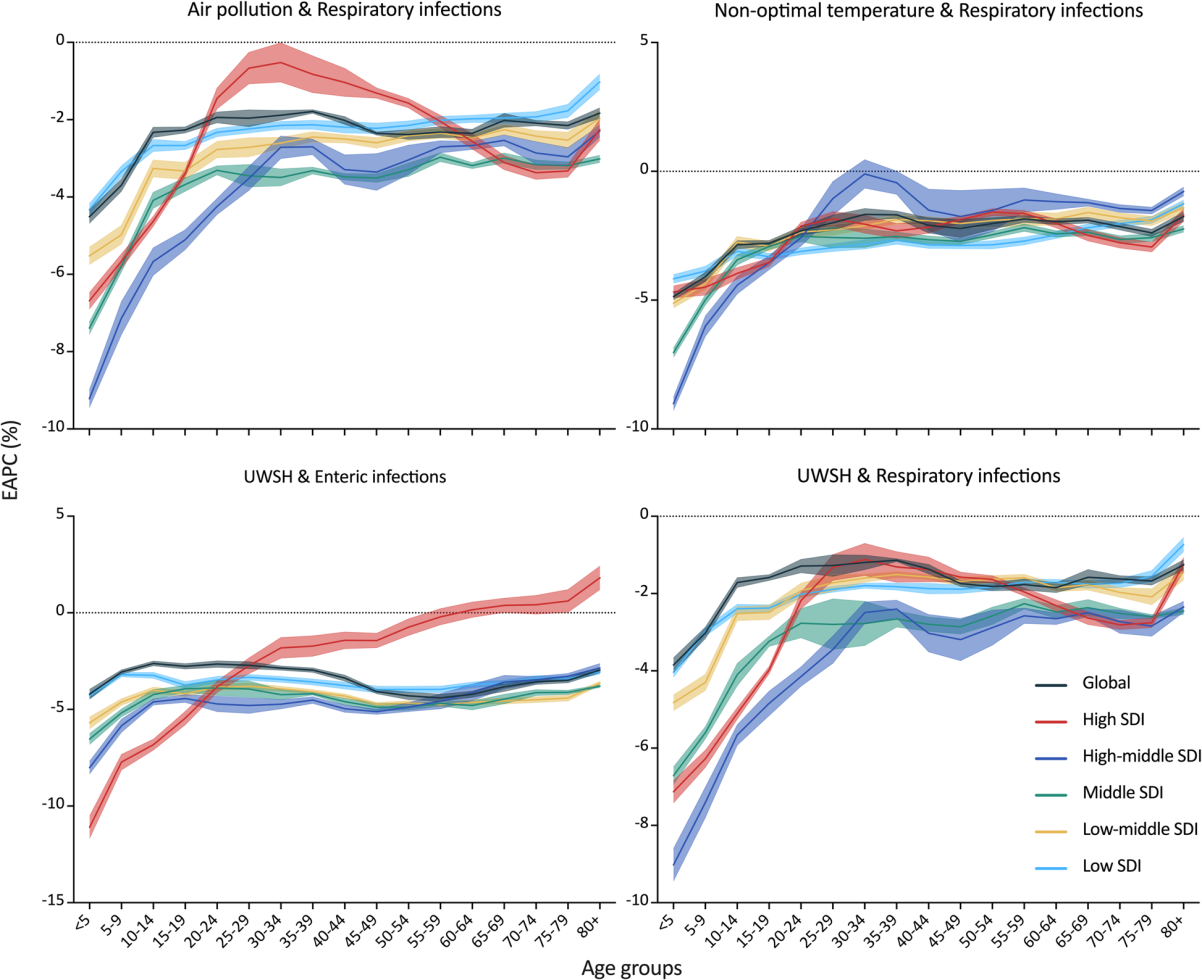
EAPC of mortality of infectious diseases attributed to air pollution, UWSH, and non-optimal temperature in different age groups, from 1990 to 2019, globally and by SDI regions. EAPC, Estimated Annual Percentage Change; SDI, Socio-Demographic Index; UWSH, unsafe water, sanitation, and handwashing

From 1990 to 2019, most age brackets showed a downward trend in mortality rates for respiratory infections resulting from air pollution, non-optimal temperature, UWSH, along with enteric infections attributed to UWSH. Children under the age of 5 exhibited the most significant decline in all diseases and SDI regions. However, in High SDI region, there were increasing trends in mortality rates for enteric infections attributed to UWSH among individuals aged 65–69 (EAPC = 0.38%, 95% CI, 0.02% to 0.75%), 75–79 (EAPC = 0.61%, 95% CI, 0.02% to 1.20%), and over 80 years (EAPC = 1.81%, 95% CI, 1.20% to 2.42%) (Fig. 4).

### PAF of environmental risk factors for infectious disease mortality

**Fig. 5:**
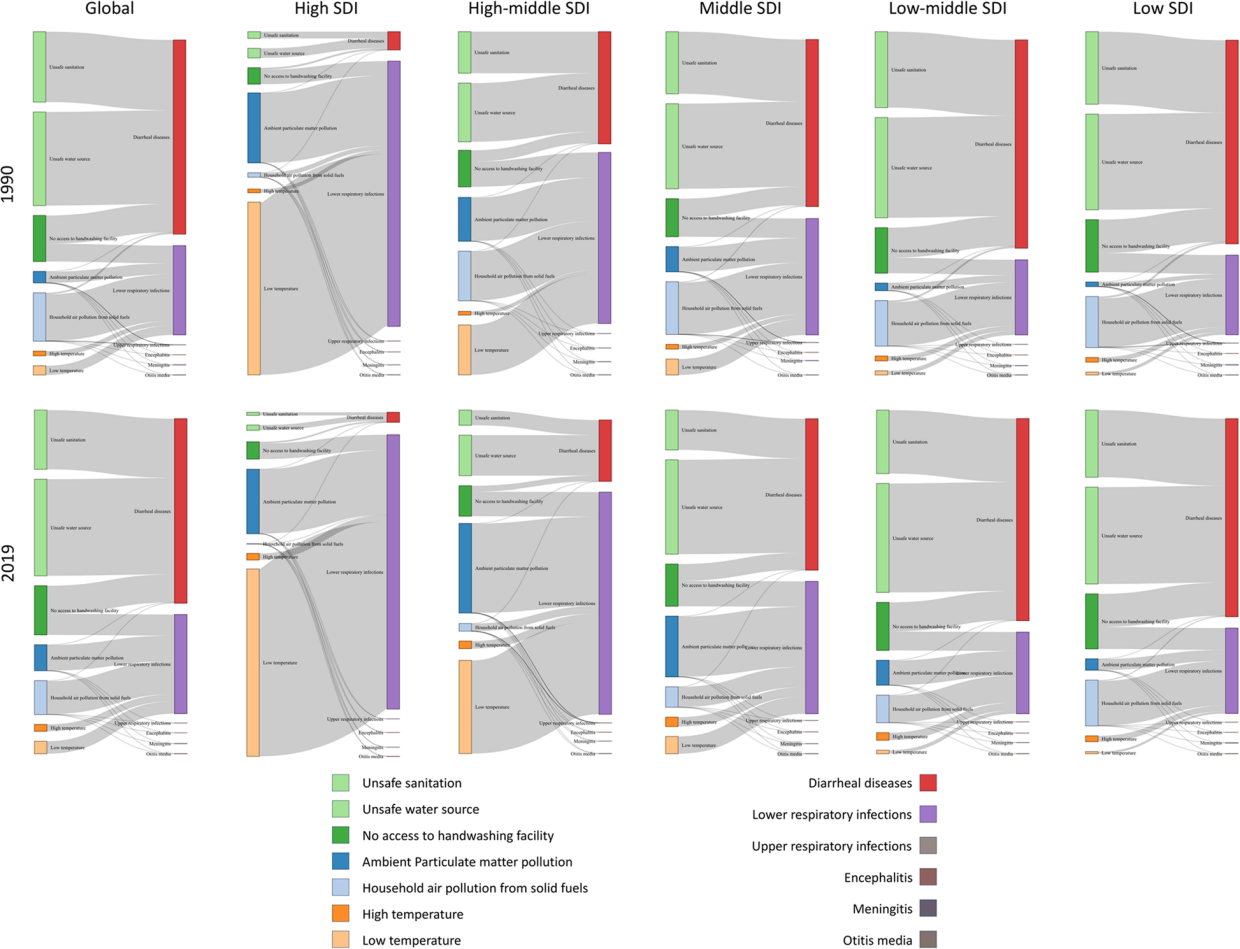
Contribution Proportion of air pollution, UWSH, and non-optimal temperature to Infectious Disease Mortality, in 1990 and 2019, globally and by SDI regions. SDI, Socio-Demographic Index

On a global scale, diarrheal diseases emerged as the predominant cause of death among infectious diseases linked to environmental risk factors, with lower respiratory infections following closely. The primary contributors to diarrheal disease deaths were unsafe sanitation, an insecure water source, and lack of access to handwashing facilities. Ambient particulate matter pollution and household air pollution from solid fuel played subsidiary roles in a minority of diarrheal disease deaths. Lower respiratory infections were attributed to factors such as the absence of handwashing facilities, ambient particulate matter pollution, household air pollution from solid fuel, high temperature, and low temperature. These conditions persisted across Middle, Low-middle, and Low SDI regions. However, in High and High-middle SDI regions, lower respiratory infections accounted for the majority of infectious disease deaths (Fig. 5).

Examining the global scenario, for deaths related to diarrheal diseases, the highest PAF in 2019 was associated with an unsafe water source (PAF = 80.17%), followed by unsafe sanitation (49.31%), and lack of access to handwashing facilities (23.32%). Concerning lower respiratory infections, household air pollution stemming from solid fuels had the highest PAF (16.96% in 2019), followed by ambient particulate matter pollution (13.09%), and the absence of handwashing facilities (10.83%). Moreover, the PAF of household air pollution played a central role in lower respiratory infections across Middle, Low-middle, and Low SDI regions. In contrast, High and High-middle SDI regions were primarily impacted by low temperature (17.43% in High SDI region and 11.77% in High-middle SDI region, in 2019). Notably, the PAF of ambient particulate matter pollution for lower respiratory infections exhibited an upward trend from 1990 to 2019. This trend was consistent in Middle (from 13.10% to 16.77%), Low-middle (from 7.62% to 18.16%), and Low (from 4.79% to 10.33%) SDI regions. Similarly, the PAF of high temperature for lower respiratory infections also exhibited an upward trajectory in these three SDI regions, rising from 2.50% to 2.61%, 5.12% to 5.58%, and 5.09% to 5.41%, from 1990 to 2019 respectively (Table 2).

**Table 2:** PAF of air pollution, UWSH, and non-optimal temperature for infectious disease mortality, in 1990 and 2019, globally and by SDI regions

Risk factors and diseases	Population Attributable Fraction (%)					
	Global	High SDI	High-middle SDI	Middle SDI	Low-middle SDI	Low SDI
***Ambient particulate matter pollution***						
Diarrheal diseases	0.25	0.13	0.50	0.52	0.22	0.14
	0.21	0.01	0.19	0.33	0.21	0.18
Encephalitis	0.09	0.06	0.14	0.16	0.05	0.06
	0.15	0.03	0.09	0.19	0.14	0.18
Lower respiratory infections	8.75	7.67	12.25	13.10	7.62	4.79
	13.09	5.96	11.27	16.77	18.16	10.33
Meningitis	0.23	0.22	0.36	0.38	0.20	0.14
	0.37	0.08	0.25	0.50	0.46	0.31
Otitis media	0.44	0.12	0.33	0.22	0.51	0.54
	0.72	0.02	0.07	0.08	0.51	1.12
Upper respiratory infections	0.09	0.03	0.13	0.14	0.06	0.02
	0.08	0.02	0.04	0.18	0.09	0.04
***Household air pollution from solid fuels***						
Diarrheal diseases	1.42	0.10	0.43	0.76	1.50	1.72
	0.47	0.00	0.02	0.07	0.28	0.80
Encephalitis	0.32	0.01	0.15	0.27	0.32	0.53
	0.17	0.00	0.01	0.04	0.15	0.52
Lower respiratory infections	36.57	0.48	13.89	27.59	45.99	53.93
	16.96	0.04	0.96	5.65	20.12	41.56
Meningitis	1.48	0.02	0.34	0.77	1.38	2.20
	1.05	0.00	0.02	0.19	0.63	1.56
Otitis media	5.08	0.00	0.28	0.65	3.65	11.69
	7.29	0.00	0.01	0.05	1.50	12.69
Upper respiratory infections	0.30	0.01	0.19	0.33	0.41	0.31
	0.14	0.00	0.01	0.04	0.13	0.27
***High temperature***						
Lower respiratory infections	3.68	0.41	1.02	2.50	5.12	5.09
	3.54	0.60	0.95	2.61	5.58	5.41
***Low temperature***						
Lower respiratory infections	7.24	18.96	14.12	8.50	4.38	3.23
	6.38	17.43	11.77	4.81	2.61	1.96
***Unsafe water source***						
Diarrheal diseases	84.32	37.14	75.46	77.81	85.37	87.08
	80.17	6.27	56.19	69.25	82.52	85.67
***Unsafe sanitation***						
Diarrheal diseases	63.41	26.11	53.40	56.75	64.63	66.03
	49.31	3.63	20.98	29.26	48.20	59.58
***No access to handwashing facility***						
Diarrheal diseases	25.50	7.17	14.54	16.87	25.64	30.42
	23.32	1.94	7.71	12.34	22.12	29.33
Lower respiratory infections	13.9	1.60	7.12	10.42	16.22	21.03
	10.83	1.45	3.10	7.07	13.99	20.46

Notes: *PAF* Population attributable fraction, *SDI* Socio-Demographic Index; For each of the risk factors/diseases, the first row shows data from 1990, and the second row shows data from 2019

### Association between SDI and infectious disease mortality attributed to air pollution, UWSH, and non-optimal temperature in 21 GBD regions

For enteric infections attributed to air pollution and UWSH, as well as other infectious diseases attributed to air pollution, and respiratory infections attributed to air pollution and UWSH, there was a consistent decrease in the ASMR of infectious diseases with the rise in SDI. The second order polynomial regression revealed a strong correlation (R-squared ranging from 0.78 to 0.91). Nonetheless, for respiratory infections triggered by non-optimal temperatures, the association between ASMR and SDI lacked statistical significance (R-squared of the second order polynomial regression was lower than 0.1).

**Fig. 6:**
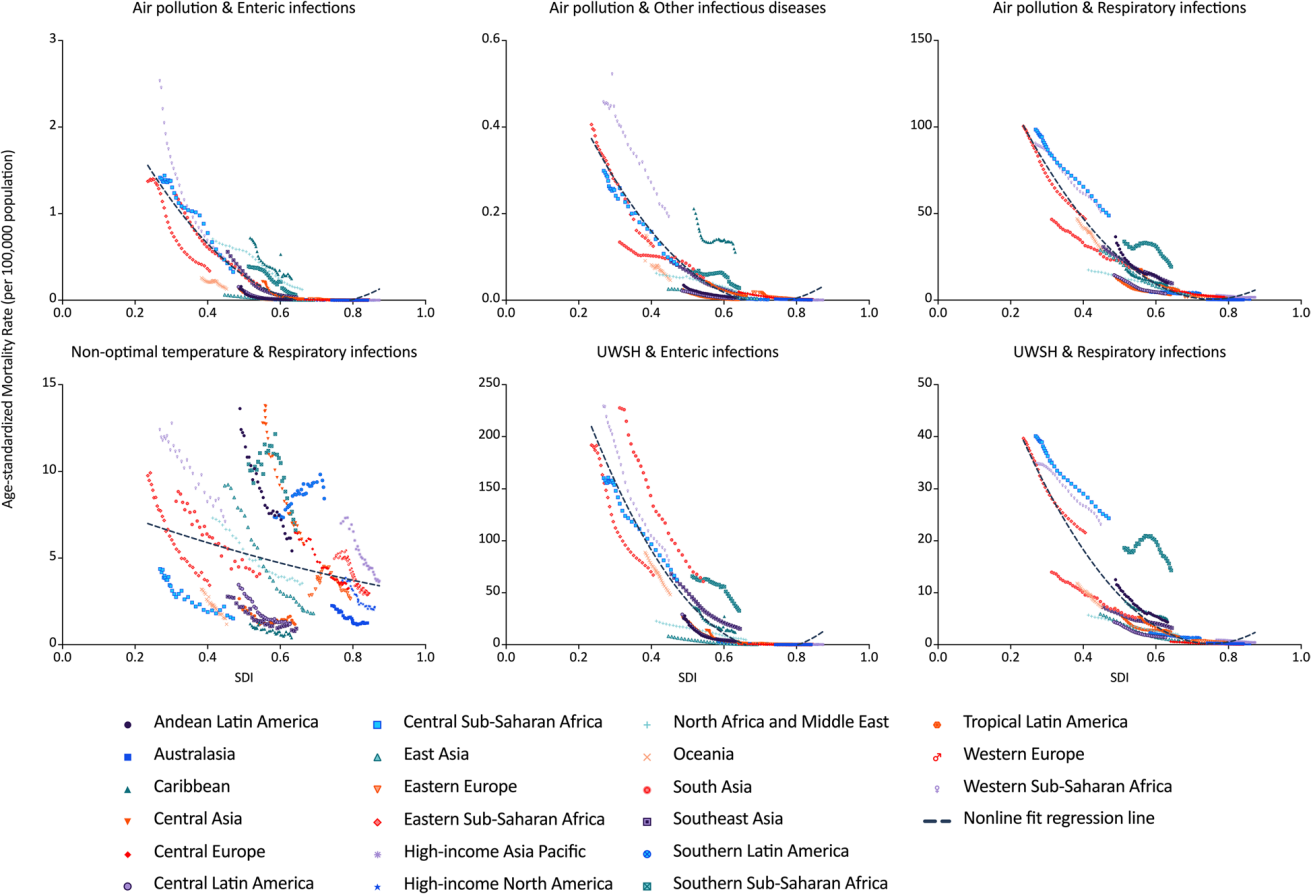
The correlation between ASMR of infectious diseases attributed to air pollution, UWSH, and non-optimal temperature and SDI in 21 GBD regions. ASMR, Age-standardized mortality rate; SDI, Socio-Demographic Index; GBD, Global Burden of Disease Study; UWSH, unsafe water, sanitation, and handwashing

On analyzing particular diseases, the Caribbean displayed the highest ASMR for enteric infections and other infectious diseases resulting from air pollution, surpassing other regions with similar SDIs. In contrast, Central, Western, and Southern Sub-Saharan Africa reported the highest ASMR for respiratory infections attributed to air pollution and UWSH compared to other regions with comparable SDIs. Additionally, South Asia recorded the highest ASMR for enteric infections attributed to UWSH when compared to regions with similar SDIs (Fig. 6).

## Discussion

To the best of our knowledge, this study represented the first comprehensive effort to outline the current burden of infectious disease mortality attributed to air pollution, UWSH, and non-optimal temperature, both globally and within various SDI regions, encompassing the estimation of its longitudinal trends over the past three decades. In summary, the analysis highlights the significant impact of certain environmental risk factors on global infectious disease mortality. Respiratory infections attributed to air pollution and enteric infections attributed to UWSH were particularly noteworthy, causing a substantial number of deaths globally, with the Low SDI region recording the highest infectious disease deaths. Both children under five years old and the elderly bore the greatest impact from air pollution, UWSH, and non-optimal temperature. While overall mortality rates showed a declining trend from 1990 to 2019, an increasing trend was observed in the High SDI region in mortality rates for enteric infections attributed to UWSH among individuals aged 65–69, 75–79, and over 80 years. Interestingly, non-optimal temperatures served as the main causes for respiratory infection deaths in High and High-middle SDI regions, while household air pollution was a fundamental contributor in lower respiratory infections across Middle, Low-middle, and Low SDI regions. Furthermore, specific infectious diseases exhibited higher mortality rates in the Caribbean, Sub-Saharan Africa (especially Central, Western, and Southern regions), and South Asia. A thorough understanding of these intricate trends and geographical variations is essential in crafting targeted interventions and policies to mitigate the impact of environmental risk factors on infectious disease mortality.

In our study, we found that respiratory infections attributed to air pollution was particularly noteworthy, contributing to 749,254 fatalities globally in 2019. This finding underscores the previously acknowledged contribution of air pollution to one in eight deaths from all diseases, emphasizing the substantial impact on infectious disease mortality [2]. Inhalation of combustion-derived material has been shown to heighten vulnerability to airway infections, as evidenced by Logan's seminal Lancet paper reporting a threefold increase in pneumonia-related deaths during the 1952 London smog, particularly affecting the very young and elderly [21]. Pneumonia notifications surged 1.4-fold during the smog and 2.4- to 2.7-fold in the following two weeks compared to the 1947–1951 weekly average. Bell et al. estimated pneumonia as a significant cause of the 12,000 excess deaths from the 1952 London smog [22]. Additionally, various combustion sources, such as environmental tobacco smoke in Vietnam, have been associated with a 1.5-fold increased risk for childhood pneumonia, with 28% of cases attributable to environmental tobacco smoke [23]. Mechanistic evidence was also emerging for bacterial pneumonia, with urban PM found to enhance pneumococcal adhesion by boosting platelet-activating factor receptor expression on airway epithelial cells, and NO_2_ exposure increasing the expression of the rhinovirus entry receptor in nasal epithelial cells [24, 25]. However, the precise impact of PM and NO_2_ on RSV infection remains uncertain [26]. Moving forward, policies aimed at reducing air pollution and mitigating its adverse health effects, particularly targeting vulnerable populations, are imperative to combating the burden of infectious diseases associated with air pollution.

In addition to air pollution, our study highlights the impact of non-optimal temperature on mortality from lower respiratory infections, particularly in High and High-middle SDI regions. Previous research has demonstrated that low temperatures were the second leading risk factor for respiratory infection deaths in America in 2019, accounting for 15.3% of fatalities, with smoking as the first leading risk factor [27]. Moreover, studies have shown that a 1 °C increase in maximum temperature was associated with a 4.2% and 3.4% increase in hospital admissions for acute lower respiratory infections among children aged 3–5 years during the dry and rainy seasons, respectively [28]. While cold effects predominate in most regions, areas with high prevailing temperatures can experience substantial heat-related effects that far exceed the burden attributable to cold temperatures [3]. Non-optimal temperatures can significantly impact respiratory infections by influencing the formation and development of microbial biofilms, a major factor in respiratory tract infection pathologies, whereby the growth and virulence of pathogenic microorganisms such as *Streptococcus pneumoniae* and nontypeable *Haemophilus* influenzae can be enhanced under such stressed conditions, further promoting the recurrence and chronicity of diseases [29, 30]. Given these findings, it is imperative to implement strategies aimed at mitigating the adverse health impacts of temperature extremes. This may include initiatives to improve urban planning and infrastructure to mitigate the urban heat island effect and enhance resilience to extreme temperatures. Additionally, targeted public health interventions and awareness campaigns could help vulnerable populations better adapt to temperature extremes and reduce their susceptibility to respiratory infections.

Moreover, we found that the PAF of ambient particulate matter pollution and high temperature for lower respiratory infections exhibited an upward trend from 1990 to 2019, in Middle, Low-middle, and Low SDI regions. In areas with lower SDI, potential issues such as inadequate public health facilities, malnutrition, and low awareness of preventive measures among residents make these populations more susceptible to the impacts of environmental particulate matter pollution and high temperatures. This might be a contributing factor to the observed phenomenon. In one study, Zhao and his team found a clear association between particulate matter pollution and respiratory diseases in Dongguan, China [31]. High temperatures are another major factor causing an increase in the rate of respiratory infections. A research study by Horne et al. stated that a rise in the concentration of particulate pollution exacerbateed the effects of weather on acute lower respiratory infections [32]. From these studies, it can be inferred that in low SDI areas, the PAF of lower respiratory infections caused by environmental particulate matter pollution and high temperatures shows an upward trend, reflecting the effects of these factors and regional characteristics on population health.

Enteric infection deaths, primarily stemming from diarrheal diseases and attributed to UWSH, were observed to be of concern across all SDI regions in our study. Even in the High SDI region, there were discernible increasing trends in mortality rates for enteric infections attributed to UWSH among individuals aged 65–69, 75–79, and over 80 years. Diarrhea could have a devastating effect on quality of life in the elderly, and the impact of diarrhea might be more pronounced in the elderly due to various causes, such as age-related structural and functional intestinal changes, consume of preventive and therapeutic drugs, compromised nutrition and hydration to withstand the effect of diarrhea, more frequent hospital admissions and courses of antibiotics, and more subtle clinical presentation than in younger patients [33]. Moreover, in nations with higher SDIs, robust health surveillance systems enable more accurate detection and documentation of diarrheal cases. However, the reliance on centralized water supply systems in these countries may also be a contributing factor. Despite being more prevalent and reliable in developed societies, these systems may still face water quality challenges, impacting diarrheal incidence [34]. Conversely, in countries with lower SDIs, the risk of diarrheal transmission may escalate due to insufficient sanitation facilities and limited hygiene education. Water quality issues, even with access to basic water supply, could contribute to diarrheal outbreaks if the water becomes contaminated. For instance, in many African cities, anthropogenic contamination of groundwater often arises from industrial discharge and untreated sewage [35]. Ensuring safe and readily available water is crucial for public health, whether for drinking, domestic use, and food production, or recreational activities. Improving water supply and sanitation, alongside enhanced management of water resources, can not only enhance economic growth but also significantly contribute to poverty reduction [36]. The multifaceted benefits of prioritizing safe water management underscore the interconnectedness of water-related initiatives with broader socio-economic goals, highlighting the pivotal role of water resources in advancing overall community well-being.

Children under 5 and the elderly, were disproportionately affected. Globally, acute lower respiratory infection remains one of the leading causes of morbidity and mortality in children younger than 5 years [37]. Antenatal exposure to air pollution may increase infants' vulnerability to respiratory infections [38]. This vulnerability could be due to underdeveloped lungs in infants with lower birth weight [39]. Postnatal lung development is crucial, with early insults potentially having lasting impacts [40]. In a Czech Republic study monitoring 1,130 children for 4.5 years, a 30% higher risk of bronchitis was found in children under 2 for every 25 mg/m^3^ increase in 30-day average PM2.5 [41]. A recent meta-analysis of 10 European birth cohorts associated PM10 and traffic exposure with increased pneumonia risk in 16,059 children across six countries [42]. While the specific timing of pollution exposure wasn't pinpointed, chronic exposure to traffic-derived air pollution was strongly linked to heightened childhood respiratory infection risk. Unsafe water, sanitation, and hygiene cause over 1 million infectious disease deaths annually, with children under 5 bearing a disproportionate burden [18, 43]. Diarrhea profoundly affects the elderly's quality of life, possibly exacerbated by age-related intestinal changes, medication use, nutritional challenges, frequent hospitalizations, antibiotic courses, and subtle clinical presentations [33]. To mitigate the impact of environmental factors on vulnerable populations such as children and the elderly, comprehensive protective measures should be implemented. This includes enhancing environmental protection measures for pregnant women to safeguard fetal health and improving the quality of drinking water to prevent contamination-related illnesses in both age groups. Additionally, efforts to monitor and improve air quality are essential to minimize the respiratory health risks posed by air pollution to both children and the elderly. Regular health check-ups for these vulnerable populations, along with prompt identification and management of potential infection symptoms, are crucial steps in safeguarding their overall health and well-being.

In our findings, Low SDI region recording the highest infectious disease deaths attributed to air pollution, UWSH, and non-optimal temperature. Moreover, the Caribbean, Sub-Saharan Africa (especially Central, Western, and Southern regions), and South Asia displayed higher mortality rates for enteric infections attributed to air pollution, respiratory infections attributed to air pollution and UWSH, and enteric infections attributed to UWSH. Given the significant disparities in infectious disease mortality attributed to environmental factors across different regions, strengthening international cooperation and health assistance is imperative. This could involve collaborative efforts in sharing knowledge, expertise, and resources to address the specific challenges faced by each region. Additionally, international aid programs focused on improving healthcare infrastructure, sanitation facilities, and access to clean water can play a crucial role in reducing the burden of infectious diseases attributed to environmental factors in regions with higher mortality rates. By fostering global partnerships and solidarity, we can work towards achieving better health outcomes for all populations, regardless of geographical location.

This study had some limitations. First, the use of yearly data from the GBD database may have led to misestimation of the disease burden in instances where original data were sparse or missing, as estimates were derived from models. Secondly, due to statistical constraints of the GBD results, only six individual diseases and seven individual risk factors were reported. Thirdly, our analysis was limited to regional-level findings, overlooking potential variations at the national level within each region. Fourth, our analysis is based on the GBD 2019 dataset, which does not cover the extensive changes in environmental factors and health outcomes that have potentially been influenced by the global COVID-19 pandemic. Therefore, the findings of this study may not reflect the situation after the COVID-19 onset. As such, a follow-up study with the GBD 2021 dataset is warranted to provide valuable insights into post-COVID-19 changes. However, despite these limitations, our findings underscore the critical need for regional cooperation in addressing infectious disease mortality attributable to environmental factors, serving as a vital call to action for global health initiatives.

## Conclusions

Our findings emphasize the significant role that air pollution, UWSH, and non-optimal temperature play in global infectious disease mortality. Respiratory and enteric infections attributed to air pollution and UWSH present considerable challenges, particularly for vulnerable populations such as children under five and the elderly. Addressing these challenges calls for targeted interventions and policies that focus on improving environmental quality, bolstering water and sanitation infrastructure, and controlling temperature extremes. Prioritizing the health and well-being of these vulnerable populations is crucial in reducing the burden of infectious diseases and further advancing global public health. Moreover, fortifying international cooperation is key to bridging the disparities across regions, propelling global public health endeavors, and achieving the SDGs.

## Data Availability

Data are available from the corresponding author by request.
